# Normal-State Transport Properties of Infinite-Layer Sr_1−*x*_La_*x*_CuO_2_ Electron-Doped Cuprates in Optimal- and Over-Doped Regimes

**DOI:** 10.3390/nano12101709

**Published:** 2022-05-17

**Authors:** Pasquale Orgiani, Alice Galdi, Darrell G. Schlom, Luigi Maritato

**Affiliations:** 1 CNR-IOM, TASC Laboratory in Area Science Park, 34139 Trieste, Italy; 2 Dipartimento di Ingegneria Industriale, Università degli Studi di Salerno, 84084 Fisciano, Italy; agaldi@unisa.it (A.G.); lmaritato@unisa.it (L.M.); 3 Department of Material Science and Engineering, Kavli Institute at Cornell for Nanoscale Science, Cornell University, Ithaca, NY 14853, USA; schlom@cornell.edu

**Keywords:** superconductivity, metal-insulator-transition, electron-doped cuprates

## Abstract

Transport properties of electron-doped cuprate Sr${}_{1-x}$La${}_{x}$CuO${}_{2}$ thin films have been investigated as a function of doping. In particular, optimal- and over-doped samples were obtained by tuning the Sr:La stoichiometric ratio. Optimal-doped samples show a non-Fermi liquid behavior characterized by linear dependence of the resistivity from room temperature down to intermediate temperature (about 150–170 K). However, by approaching temperatures in the superconducting transition, a Fermi-liquid behavior-characterized by a $T^{2}$-scaling law-was observed. Once established, the transition from a linear-*T* to a quadratic-$T^{2}$ behavior was successfully traced back in over-doped samples, even occurring at lower temperatures. In addition, the over-doped samples show a crossover to a linear-*T* to a logarithmic dependence at high temperatures compatible with anti-ferromagnetic spin fluctuations dominating the normal state properties of electron-doped cuprates.

## 1. Introduction

The normal-state transport properties and their correlations with the superconducting ones are still among the most investigated aspects of the electron-doped cuprates [1,2,3]. However, differently from the hole-doped parent compounds, a conclusive drawing about the fundamental mechanisms behind their normal state is still missing. As a matter of fact, a predominant electron-electron scattering mechanism supported by a $T^{2}$-scaling law up to 250 K has been initially proposed to describe the normal metal phase of this class of materials [4]. However, a non-saturating linear temperature dependence of resistivity [5,6,7], sometimes accompanied by other *unconventional* behaviors (for example, $\rho \propto T^{1.6}$) [8,9] has questioned the validity of such modeling. In particular, such non-Fermi liquid properties have been explained in terms of the proximity of the superconducting phase with a quantum critical point, usually identified as the quantum transition to the anti-ferromagnetic phase [7,10,11,12,13,14]. In addition, in the under-doped samples, the sizable increase of the resistivity when approaching the superconducting transition affects not only the precise determination of the temperature dependence of the normal-metal phase, but also its origin remains undetermined. The metal-insulator-transition (MIT) in fact occurs for a critical quantum values of $k_{F}l\approx$ 20–25 [15], which is fairly larger than 4 and 1 reported for low- [16] and high-temperature superconductors [17], respectively. Such a puzzling scenario has thus prevented so far the full understanding of the normal-state properties of the electron-doped superconductors.

**Figure 1:**
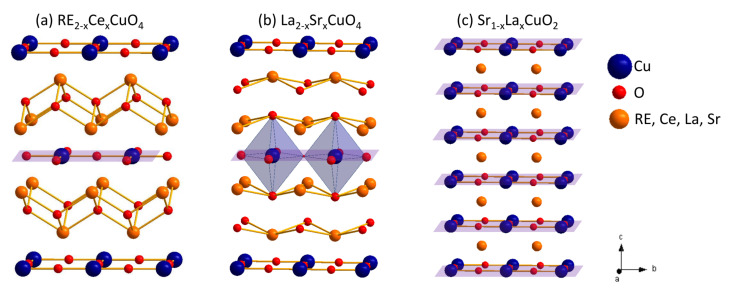
Crystal structures of electron-doped Nd${}_{2-x}$Ce${}_{x}$CuO${}_{4}$ (**a**), hole-doped La${}_{2-x}$Sr${}_{x}$CuO${}_{4}$ (**b**), and infinite-layer Sr${}_{1-x}$La${}_{x}$CuO${}_{2}$ (**c**). The superconducting CuO${}_{2}$ sheets are enlightened by magenta planes (Cu and O ions are blue and red, respectively; other ions are orange-colored).

With the aim of revealing the nature of the normal-state resistivity in electron-doped cuprates, transport properties of a wide series of infinite layer (IL) Sr${}_{1-x}$La${}_{x}$CuO${}_{2}$ (SLCO) thin films have been investigated [18]. IL compounds are only formed by CuO${}_{2}$ sheets separated by electro-positive ions (namely, Sr${}^{2+}$ and La${}^{3+}$) and are known to be the truly superconducting block [17,19]. The IL cuprate structure, as discussed here, offers unique opportunities to define the proper regime of the transport properties, having removed the ambiguity regarding the truly conducting layer [20,21,22,23,24] (Figure 1).

Our starting point was the optimal-doped regime, where localization effects are negligible [19] and a non-Fermi liquid behavior characterized by linear dependence of the resistivity was identified in the majority of the investigated temperature range (from about 150 K to 300 K). However, in the proximity of the superconducting transition, a change in the temperature dependence of the resistivity was observed and correlated to a hidden Fermi-liquid behavior characterized by a $T^{2}$-scaling law [25,26]. Once established, the transition from a linear-*T* to a quadratic-$T^{2}$ behavior was successfully traced back in over-doped samples, even though occurring at lower temperatures, thus, confirming a Fermi liquid behavior of electron-doped cuprates in proximity of the superconducting transition in a wider range of doping [27]. Moreover, as reported in the literature for both hole- [13,28,29,30] and electron-doped cuprates [5], the resistivity of over-doped samples deviates from a linear-*T* dependency towards a saturation of its value. In particular, a logarithmic dependence of resistivity well fits the experimental data and has been correlated with critical spin fluctuations originating from an anti-ferromagnetic quantum critical point [7,10,27,31]. Therefore, we are in a position to fully described the normal state properties of electron-doped cuprate systems as a function of doping and temperature [1].

## 2. Materials and Methods

SLCO films were grown on (110)-oriented GdScO${}_{3}$ substrates by Veeco GEN10 dual-chamber oxide Molecular Beam Epitaxy system using a shuttered layer-by-layer deposition process performed in purified O${}_{3}$[32,33]. In order to remove all of the apical extra-oxygens, SLCO thin films were post-annealed in UHV for 30 min at high temperatures soon after the deposition. As for many other oxide materials [34,35,36,37], such a process is well known to easily promote oxygen re-evaporation from the film. In addition, since substrate-induced tensile strain has been demonstrated to cooperate in the removal of the undesired apical oxygen ions [38,39], SLCO were routinely grown on scandiate oxide substrates RE-ScO${}_{3}$ (with RE as Rare Earth ions, such as Gd in our case), characterized by large in-plane lattice parameters [40]. Standard X-ray diffraction characterization has been routinely performed for all the deposited samples, thus confirming the IL phase (i.e., without the presence of apical oxygen, which would correspond to a sizeably larger out-of-plane lattice parameter [25,26]). The doping level and, therefore, the superconducting critical temperature *T*${}_{c}$ was varied by tuning the Sr:La stoichiometric ratio. Details on the growth process, as well as the structural characterization of the samples, are reported elsewhere [39,41,42]. Since SLCO easily degrades during photo-lithography, all transport properties reported here were measured on unpatterned films. Electrical transport measurements were carried out by a standard four-points-probe DC technique in the Van der Pauw configurations with a pulsed reverse-bias current [43].

In order to uniform the fitting procedures, with $\chi^{2}$ being affected by the number of fitted points, we kept them fixed for all curves (i.e., 1 point per K). The fitting temperature range was determined by continuously monitoring both the statistical error of fitted parameters as well as the reduced $\chi^{2}$ values ($\chi^{2}=\left(\frac{1}{N}\right)\cdot \sum_{i=1}^{N}\left[\frac{{\left(\rho_{exp}^{i}-\rho_{fit}^{i}\right)}^{2}}{{\left(\rho_{fit}^{i}\right)}^{2}}\right]$). As the fitting temperature range increases, the statistical error on the fitting parameters monotonically decreases (well below the 1%), while the reduced $\chi^{2}$ value remains substantially unchanged. However, when the theoretical formulas no longer well represent the experimental data, the statistical $\chi^{2}$ value suddenly increases (as it will be shown for over-doped samples), establishing the limit of the validity for the fitted formula [44].

## 3. Results

**Figure 2:**
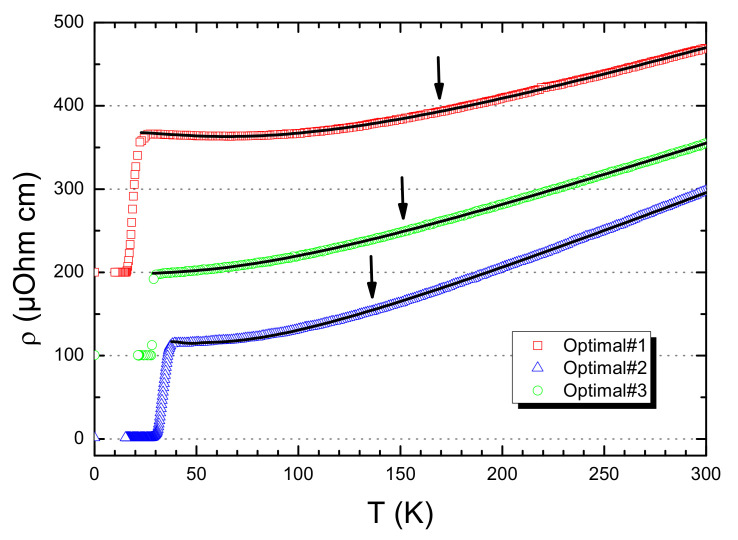
(Color online) Resistivity curves for three selected SLCO samples with an electronic doping close to the optimal condition. The best fit curves are also reported. Arrows indicate the temperatures below which the resistivity curves are no longer purely linear, namely $T_{L}$. Data are shifted 100 μOhm·cm for clarity.

Transport properties of three representative SLCO samples close to optimal-doping conditions are reported in Figure 2. As the optimal doping approaches, the resistivity curve tends to be metallic at any temperatures (i.e., Δρ/ΔT>0), though it slightly upturns in a few samples closer to the under-doping regime.

Following previous reports [25,26], in addition to the linear *T* term, we also considered a quadratic $T^{2}$ term due to Fermi-liquid behavior in the fitting formulas for resistivity curves (Equation (1)). For comparison, as reported in [4,8,9], we also used a generic $T^{n}$ power law dependence (Equation (1)):

(1) $$\rho_{metal}\left(T\right)=\rho_{0}+A_{1}\cdot T+A_{2}\cdot T^{2}$$

(2) $$\rho_{metal}\left(T\right)=\rho_{0}+A_{n}\cdot T^{n}$$

with $\rho_{0}$, $A_{1}$, $A_{2}$ and $\rho_{0}$, $A_{n}$, *n* as fitting parameters in the two cases. As successfully demonstrated for a similar strongly correlated system [45,46], the best-fit value of *n* helps to reveal the main active scattering process among different ones, ranging from the interaction with thermal as well as acoustic phonons, spin-wave scattering phenomena and others [47]. We here underline that in Equation (1), the power-law exponents *n* are fixed to 1 and 2, while it is free to vary in the Equation (2). As a consequence, the fitting parameters were three in both expressions. The statistical $\chi^{2}$ values referring to data reported in Figure 2 are summarized in Table 1.

As evident, the combination of linear *T* and quadratic $T^{2}$ terms better fits the experimental data rather than a generic $T^{n}$ power-law dependency [8,9]. In addition, while all the best-fit values for *n* cannot be trivially correlated to any known scattering process (i.e., 2.08, 1.66 m and 1.51 for the Optimal♯1, the Optimal♯2, and the Optimal♯3 samples, respectively), the addition of a quadratic correction to the residual resistivity in proximity of the superconducting temperatures well reproduces the soft flattening of the resistivity curve at low temperatures, thus further confirming the solidity of the assumption of a linear *T* behavior for the metallic term $\rho_{M}\left(T\right)$ at higher temperatures.

**Figure 3:**
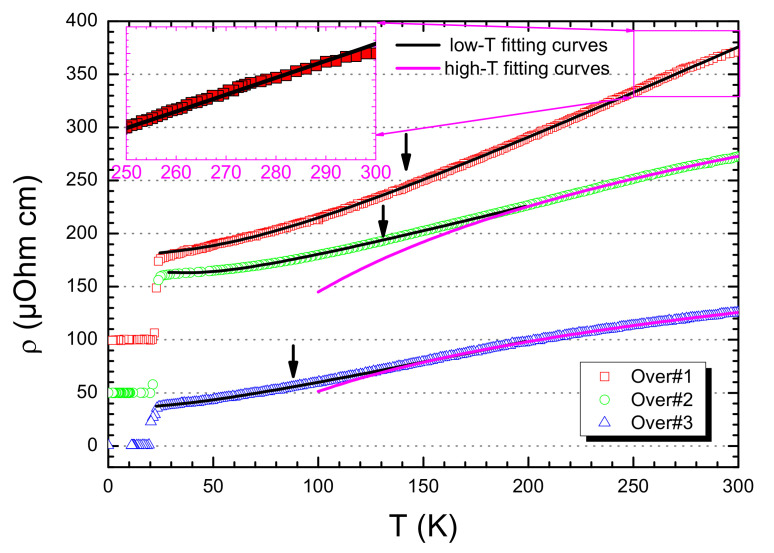
(Color online) Resistivity curves for three selected over-doped SLCO samples. In the inset (magenta box), a zooming-out of the high-temperature regime for the Over♯1 sample. For all curves, the low temperature regime has been fitted by using Equation (1) (black curves), while the high temperature regime has been fitted for the Over♯2 and Over♯3 sample by a logarithmic behavior (magenta curves). Arrows indicate the temperatures at which, at low temperatures, the resistivity curves are no longer purely linear, namely $T_{L}$. Data are shifted 50 μOhm·cm for clarity.

The same approach was followed for the SLCO over-doped samples (Figure 3). However, either using a combination of linear *T* and quadratic $T^{2}$ terms (Equation (1)) or by using a generic $T^{n}$ power law (Equation (2)), the statistical $\chi^{2}$ values were considerably larger (by falling in the range of 10${}^{-4}$) than those obtained for the optimal-doped samples. Such a feature can be directly connected to the upward concavity of the resistivity curves at high temperatures, which has been observed in a large number of cuprates superconductors [5,27,28]. As a matter of fact, it is very pronounced as the over-doping level increases (i.e., Over♯2 and Over♯3 for *T* > 230 and 210 K, respectively) and still visible in those slightly over-doped (i.e., Over♯1 for *T* > 250 K, see inset of Figure 3).

Despite the large number of reports of such deviations from linearity [5,13,24,27,28,29,48], such a transition is generally disregarded. Inelastic neutron scattering studies, on Pr${}_{0.89}$La Ce${}_{0.11}$CuO${}_{4}$[49] and similar electron-doped compounds [50], have revealed that high energy spin excitation of such compounds are similar to those observed in anti-ferromagnetic alloys. Moreover, observations of Cu spin-fluctuations have been proved by muon spin relaxation experiments in cuprate superconductors [51]. In this respect, Rivier and Zlatic developed a model describing the resistivity of itinerant electrons scattered by localized anti-ferromagnetic spin fluctuations [52] where a linear scaling-law for the resistivity only occurs up to the so-called spin-fluctuation temperature $T_{sf}$. Above such a temperature, the resistivity shows a $\rho_{sf}$ + C·ln($T/T_{sf}$) logarithmic dependence, to finally approach to an asymptotic value. Triggered by the observed sub-linear behavior of the resistivity and by finding that electron carriers in electron-doped cuprates have indeed a strong itinerant character [50], we tested the applicability of the Rivier and Zlatic model to the over-doped SLCO samples. We therefore fit the resistivity curves, by assuming a lower temperature range in which we used Equations (1) and (2), while the high temperature regime was fit by using a logarithmic scaling law. Assuming this, the agreement between theoretical prediction and experimental data was quite impressive, as demonstrated by the sizable decrease of the statistical $\chi^{2}$ values from $10^{-4}$ to $10^{-6}$, which are comparable with those previously obtained for the optimal-doped SLCO samples (data are reported in Table 2).

Analogously to the other SLCO samples, the low temperature resistivity behavior was better fit by using a combination of the linear *T* and quadratic $T^{2}$ behavior for the metallic phase rather than the generic $T^{n}$ power law dependence (Equation (2)). Moreover, similar to the optimal-doped cases, the calculated values of the *n* parameters cannot be correlated to any known scattering mechanism (namely, 1.38, 1.50, and 1.25 for Over♯1, Over♯2, and Over♯3 samples, respectively). Furthermore, within the scenario of a normal-state resistivity dominated by spin-fluctuations [52], a linear *T* dependency for the metallic state is indeed expected to be replaced by a logarithmic behavior at high temperatures. This is exactly what has been observed in over-doped SLCO samples and the best fit procedure provided values for the spin-fluctuation temperature $T_{sf}$ of about 180 K and 160 K for the Over♯2 and Over♯3 SLCO samples, respectively.

## 4. Discussion

Remarkable conclusions can be derived by our analysis. The linear *T* metallic regime of the normal-state conductivity unambiguously demonstrates the non-Fermi liquid behavior at high temperatures of electron-doped cuprates at any doping level. A combination of linear *T* and quadratic $T^{2}$ terms, describing the presence of a cross-over from a non-Fermi to a Fermi liquid behavior, is able to well fit the transport properties in the proximity of the superconducting transition.

**Figure 4:**
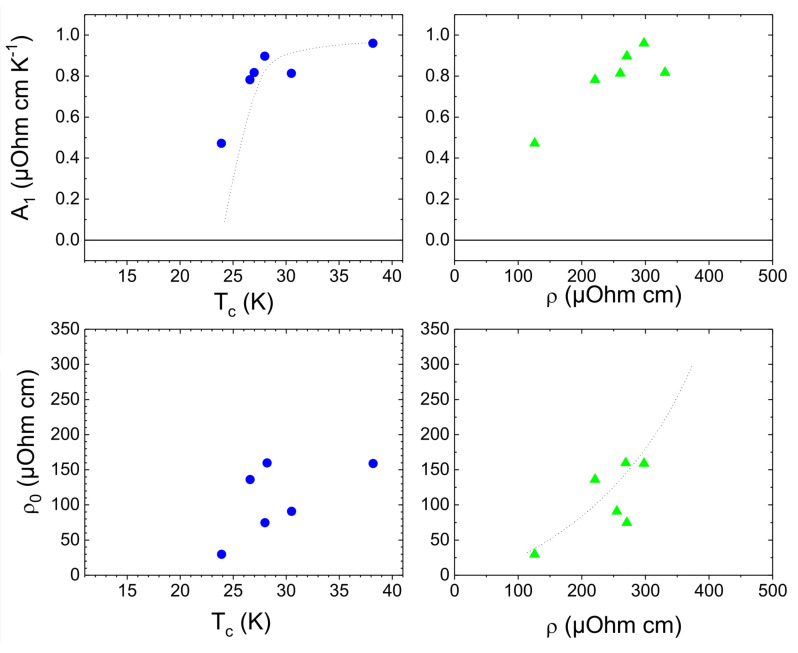
(Color online) Best-fit parameters $A_{1}$ and $\rho_{0}$ (upper and lower panels, respectively) as a function of the superconducting temperature $T_{c}$ (blue dots) and the room temperature resistivity ρ (green triangles), respectively. Lines are guides for eyes.

Moreover, further information can be derived when best-fit values of $A_{1}$ and $\rho_{0}$ (reported in Table 3) are plotted in term of two physical quantities related to the superconducting and the normal state: namely, the superconducting transition temperature $T_{c}$ and the room temperature resistivity ρ, respectively (Figure 4).

The evolution of the scattering coefficient $A_{1}$, obtained from the linear *T* term in the resistivity equation, surprisingly revealed a direct correlation with the superconducting temperature $T_{c}$ (therefore, with the superconducting phase) rather than the room temperature resistivity ρ (top panels in Figure 4) or the doping level, as previously reported [27,30]. More in detail, $A_{1}$ monotonically increased with $T_{c}$ by reaching the maximum value of about 1 μOhm·cm·K${}^{-1}$ in an optimal-doped sample and subsequently reduced in the over-doped regime. Our previous analysis [19] provided evidence that the $A_{1}$ coefficient also approaches zero in the under-doped regime, thus enlightening a profound relationship between $T_{c}$ and the strength of the linear-temperature inelastic scattering coefficient $A_{1}$.

Conversely, the best-fit values of the residual resistivity $\rho_{0}$ (as defined in Equations (1) and (2)) show a strong dependency on the normal state properties rather than the superconducting ones. This is clearly evident by analyzing the lower panels of Figure 4. As the room temperature resistivity ρ decreases, the residual resistivity $\rho_{0}$ also monotonically decreases, by reaching a limit value of about 30 μOhm·cm. However, the apparent random distribution of the $\rho_{0}$ values as a function of the superconducting critical temperature $T_{c}$ reveals profound differences between electron doped cuprates and conventional BCS-superconductors, where a strong dependency of the $T_{c}$ values as a function of the residual resistivity $\rho_{0}$ and/or the residual resistivity ratio RRR (defined as $\rho /\rho_{0}$) has been widely reported [54,55].

## 5. Conclusions

**Figure 5:**
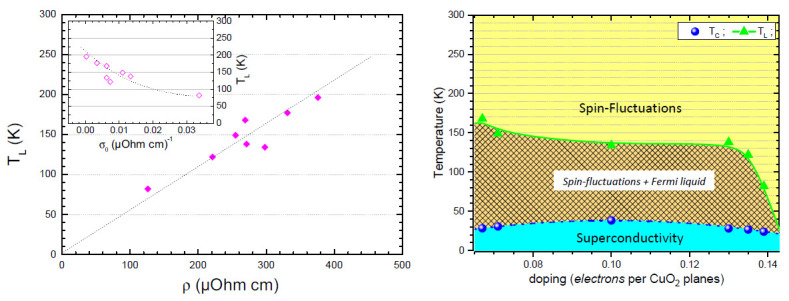
**Left** panel—$T_{L}$ dependence on the room temperature resistivity ρ. In the inset, $T_{L}$ is also plotted as a function of the residual conductivity $\sigma_{0}$. **Right** panel—Doping evolution of the temperature phase diagram of electron doped cuprates. The experimental values of the superconducting temperature $T_{c}$ and the temperature $T_{L}$ at which the curves are no longer purely linear are reported. The different regimes are qualitatively indicated by colors and labels.

Our analysis provides evidence that a non-Fermi liquid behavior correlated to a spin-fluctuation regime dominates the normal state transport properties of electron-doped SLCO thin films. However, by approaching the superconducting phase, a quadratic-$T^{2}$ Fermi-liquid behavior is established in both optimal- and over-doped SLCO samples, though being reduced as the doping level increases. By indicating with $T_{L}$ the temperature at which correction to a linear *T* scaling law becomes relevant, a strong correlation with the room temperature resistivity ρ can be found (left panel in Figure 5). Such a quantity ultimately determines the temperature at which the transition from a Fermi/non-Fermi liquid behavior occurs.

Finally, in over-doped SLCO samples, a transition from a linear *T* to a log *T* scaling-law has been observed and tentatively correlated to the high-temperature regime of itinerant electrons scattered by localized anti-ferromagnetic spin fluctuations. The linear *T* non-Fermi liquid behavior of resistivity has been therefore proved to be a common feature of both electron doped and hole doped cuprates at high temperatures, suggesting a symmetry between the hole- and electron-doped phase diagrams. Hence, by estimating the expected charge carrier concentration $n_{exp}$ from the superconducting critical temperature $T_{c}$ [53], a phase-diagram can be therefore derived (right panel in Figure 5). Such a symmetry may be limited to the infinite layer case or extend to all electron doped compounds, despite the many differences arising between the two classes of materials.

## Figures and Tables

**Table 1:** Normalized $\chi^{2}$ values for three selected SLCO samples with electronic doping close to the optimal condition (data refer to samples reported in Figure 2) for the fitting formulas in Equations (1) and (2).

Sample	$\rho_{\mathrm{power}-\mathrm{law}}=\rho_{0}+A_{n}\cdot T^{n}$	$\rho_{\mathrm{linear}}+\rho_{\mathrm{quadratic}}=\rho_{0}+A_{1}\cdot T+A_{2}\cdot T^{2}$
	(10${}^{-5}$)	(10${}^{-5}$)
Optimal♯1	6.7	0.21
Optimal♯2	4.7	1.4
Optimal♯3	2.5	0.15

**Table 2:** Normalized $\chi^{2}$ values for three selected over-doped SLCO samples. Data refer to samples reported in Figure 3. The low temperature fitting range for Over♯2 and Over♯3 samples is up to 230 K and 210 K, respectively.

Sample	$T_{\mathrm{sf}}$	$\rho_{\mathrm{power}-\mathrm{law}}=\rho_{0}+A_{n}\cdot T^{n}$	$\rho_{\mathrm{linear}}+\rho_{\mathrm{quadratic}}=\rho_{0}+A_{1}\cdot T+A_{2}\cdot T^{2}$
	(K)	(10${}^{-5}$)	(10${}^{-5}$)
Over♯1	—	2.39	0.65
Over♯2	180	0.97	0.16
Over♯3	160	1.30	0.48

**Table 3:** The superconducting transition $T_{c}$ temperatures, the expected electronic doping $n_{exp}$ estimated from the superconducting critical temperatures $T_{c}$ [53] and the $T_{L}$ defined as the temperature at which resistivity curves are no longer linear. The best fit parameters $A_{1}$ and $\sigma_{0}$ are also reported.

Sample	$T_{c}$	$n_{\exp}$	$T_{L}$	$\rho_{0}$	$A_{1}$
	(K)		(K)	(μOhmcm)	$\left(\mu {\mathrm{Ohm}\;\mathrm{cm}\;K}^{-1}\right)$
Optimal♯1	28.2	0.067	168	160	0.81
Optimal♯2	30.5	0.071	149	91	0.81
Optimal♯3	38.2	0.100	134	157	0.96
Over♯1	28.2	0.130	138	75	0.89
Over♯2	26.6	0.135	122	136	0.78
Over♯3	23.9	0.139	82	30	0.47

## Data Availability

The data presented in this study are available on request from the corresponding author.

## References

[1] Armitage N.P., Fournier P., Greene R.L. (2010). Progress and perspectives on electron-doped cuprates. Rev. Mod. Phys..

[2] Li Y., Tabis W., Tang Y., Yu G., Jaroszynski J., Barišić N., Greven M. (2019). Hole pocket–driven superconductivity and its universal features in the electron-doped cuprates. Sci. Adv..

[3] Sarkar T., Mandal P.R., Poniatowski N.R., Chan M.K., Greene R.L. (2019). Correlation between scale-invariant normal-state resistivity and superconductivity in an electron-doped cuprate. Sci. Adv..

[4] Tsuei C., Gupta A., Koren G. (1989). Quadratic temperature dependence of the in-plane resistivity in superconducting Nd_1.85_CuO_4_—Evidence for Fermi-liquid normal state. Phys. C Supercond..

[5] Charpentier S., Roberge G., Godin-Proulx S., Béchamp-Laganière X., Truong K.D., Fournier P., Rauwel P. (2010). Antiferromagnetic fluctuations and the Hall effect of electron-doped cuprates: Possibility of a quantum phase transition at underdoping. Phys. Rev. B.

[6] Higgins J.S., Dagan Y., Barr M.C., Weaver B.D., Greene R.L. (2006). Role of oxygen in the electron-doped superconducting cuprates. Phys. Rev. B.

[7] Jin K., Zhang X.H., Bach P., Greene R.L. (2009). Evidence for antiferromagnetic order in La_2−*x*_Ce_*x*_CuO_4_ from angular magnetoresistance measurements. Phys. Rev. B.

[8] Butch N.P., Jin K., Kirshenbaum K., Greene R.L., Paglione J. (2012). Quantum critical scaling at the edge of Fermi liquid stability in a cuprate superconductor. Proc. Natl. Acad. Sci. USA.

[9] Jin K., Butch N.P., Kirshenbaum K., Paglione J., Greene R.L. (2011). Link between spin fluctuations and electron pairing in copper oxide superconductors. Nature.

[10] Lyons K.B., Fleury P.A. (1988). Spin fluctuations in superconducting cuprates. J. Appl. Phys..

[11] Mirarchi G., Seibold G., Di Castro C., Grilli M., Caprara S. (2022). The strange-metal behavior of cuprates. Condens. Matter.

[12] Moriya T., Ueda K. (2000). Spin fluctuations and high temperature superconductivity. Adv. Phys..

[13] Rice T.M., Robinson N.J., Tsvelik A.M. (2017). Umklapp scattering as the origin of T-linear resistivity in the normal state of high-T_c_ cuprate superconductors. Phys. Rev. B.

[14] Taillefer L. (2010). Scattering and pairing in cuprate superconductors. Annu. Rev. Condens. Matter Phys..

[15] Fournier P., Mohanty P., Maiser E., Darzens S., Venkatesan T., Lobb C.J., Czjzek G., Webb R.A., Greene R.L. (1998). Insulator-Metal crossover near optimal doping in Pr_2−*x*_Ce_*x*_CuO_4_: Anomalous normal-state low temperature resistivity. Phys. Rev. Lett..

[16] Liu Y., Haviland D.B., Nease B., Goldman A.M. (1993). Insulator-to-superconductor transition in ultrathin films. Phys. Rev. B.

[17] Orgiani P., Aruta C., Balestrino G., Born D., Maritato L., Medaglia P.G., Stornaiuolo D., Tafuri F., Tebano A. (2007). Direct measurement of sheet resistance R_□_ in cuprate systems: Evidence of a Fermionic scenario in a metal-insulator transition. Phys. Rev. Lett..

[18] Shaked H. (1994). Crystal Structures of the High-Tc Superconducting Copper-Oxides.

[19] Orgiani P., Galdi A., Sacco C., Arpaia R., Charpentier S., Lombardi F., Barone C., Pagano S., Schlom D.G., Maritato L. (2015). The role of Quantum Interference Effects in normal-state transport properties of electron-doped cuprates. J. Supercond. Nov. Magn..

[20] Mandrus D., Forro L., Kendziora C., Mihaly L. (1991). Two-dimensional electron localization in bulk single crystals of Bi_2_Y_*x*_Ca_1−*x*_Cu_2_O_8_. Phys. Rev. B.

[21] Tanda S., Honma M., Nakayama T. (1991). Critical sheet resistance observed in high-*T*_c_ oxide-superconductor Nd_2−*x*_Ce_2_CuO_4_. Phys. Rev. B.

[22] Seidler G.T., Rosenbaum T.F., Veal B.W. (1992). Two-dimensional superconductor-insulator transition in bulk single-crystal YBa_2_Cu_3_O_6.38_. Phys. Rev. B.

[23] Arpaia R., Golubev D., Baghdadi R., Ciancio R., Dražić G., Orgiani P., Montemurro D., Bauch T., Lombardi F. (2017). Transport properties of ultrathin YBa_2_Cu_3_O_7−*δ*_ nanowires: A route to single-photon detection. Phys. Rev. B.

[24] Jovanović V.P., Li Z.Z., Raffy H., Briatico J., Sinchenko A.A., Monceau P. (2009). Resistive upper critical fields and anisotropy of an electron-doped infinite-layer cuprate. Phys. Rev. B.

[25] Sacco C., Galdi A., Orgiani P., Coppola N., Wei H.I., Arpaia R., Charpentier S., Lombardi F., Goodge B., Kourkoutis L.F. (2019). Low temperature hidden Fermi-liquid charge transport in under doped La_*x*_Sr_1−*x*_CuO_2_ infinite layer electron-doped thin films. J. Phys. Condens. Matter.

[26] Sacco C., Galdi A., Romeo F., Coppola N., Orgiani P., Wei H.I., Goodge B.H., Kourkoutis L.F., Shen K., Schlom D.G. (2019). Carrier confinement effects observed in the normal-state electrical transport of electron-doped cuprate trilayers. J. Phys. D Appl. Phys..

[27] Barisic N., Chan M.K., Li Y., Yu G., Zhao X., Dressel M., Smontara A., Greven M. (2013). Universal sheet resistance and revised phase diagram of the cuprate high-temperature superconductors. Proc. Natl. Acad. Sci. USA.

[28] Poniatowski N.R., Sarkar T., Das Sarma S., Greene R.L. (2021). Resistivity saturation in an electron-doped cuprate. Phys. Rev. B.

[29] Arouca R., Marino E.C. (2021). The resistivity of high-T_*c*_ cuprates. Supercond. Sci. Technol..

[30] Pelc D., Veit M.J., Dorow C.J., Ge Y., Barišić N., Greven M. (2020). Resistivity phase diagram of cuprates revisited. Phys. Rev. B.

[31] Hussey N.E. (2008). Phenomenology of the normal state in-plane transport properties of high-T_*c*_ cuprates. J. Phys. Condens. Matter.

[32] Schlom D.G., Chen L.Q., Pan X., Schmehl A., Zurbuchen M.A. (2008). A Thin Film Approach to Engineering Functionality into Oxides. J. Am. Ceram. Soc..

[33] Schlom D., Haeni J., Lettieri J., Theis C., Tian W., Jiang J., Pan X. (2001). Oxide nano-engineering using MBE. Mater. Sci. Eng. B.

[34] Orgiani P., Petrov A.Y., Ciancio R., Galdi A., Maritato L., Davidson B.A. (2012). Evidence of direct correlation between out-of-plane lattice parameter and metal-insulator transition temperature in oxygen-depleted manganite thin films. Appl. Phys. Lett..

[35] Bigi C., Tang Z., Pierantozzi G.M., Orgiani P., Das P.K., Fujii J., Vobornik I., Pincelli T., Troglia A., Lee T.L. (2020). Distinct behavior of localized and delocalized carriers in anatase TiO_2_ (001) during reaction with O_2_. Phys. Rev. Mater..

[36] Gobaut B., Orgiani P., Sambri A., di Gennaro E., Aruta C., Borgatti F., Lollobrigida V., Céolin D., Rueff J.P., Ciancio R. (2017). Role of oxygen deposition pressure in the formation of Ti defect states in TiO_2_ (001) anatase thin films. ACS Appl. Mater. Interfaces.

[37] Orgiani P., Adamo C., Barone C., Galdi A., Pagano S., Petrov A.Y., Quaranta O., Aruta C., Ciancio R., Polichetti M. (2008). Epitaxial growth of La_0.7_Ba_0.3_MnO_3_ thin films on MgO substrates: Structural, magnetic, and transport properties. J. Appl. Phys..

[38] Karimoto S., Naito M. (2004). Electron-doped infinite-layer thin films with $T_{c}^{\mathrm{zero}}$ = 41 *K* grown on DyScO_3_ substrates. Phys. C Supercond..

[39] Galdi A., Orgiani P., Sacco C., Gobaut B., Torelli P., Aruta C., Brookes N.B., Minola M., Harter J.W., Shen K.M. (2018). X-ray absorption spectroscopy study of annealing process on Sr_1−*x*_La_*x*_CuO_2_ electron-doped cuprate thin films. J. Appl. Phys..

[40] Uecker R., Bertram R., Brützam M., Galazka Z., Gesing T.M., Guguschev C., Klimm D., Klupsch M., Kwasniewski A., Schlom D.G. (2017). Large-lattice-parameter perovskite single-crystal substrates. J. Cryst. Growth.

[41] Harter J.W., Maritato L., Shai D.E., Monkman E.J., Nie Y., Schlom D.G., Shen K.M. (2012). Nodeless superconducting phase arising from a strong (*π*, *π*) antiferromagnetic phase in the infinite-layer electron-doped Sr_1−*x*_La_*x*_CuO_2_ Compound. Phys. Rev. Lett..

[42] Maritato L., Galdi A., Orgiani P., Harter J.W., Schubert J., Shen K.M., Schlom D.G. (2013). Layer-by-layer shuttered molecular-beam epitaxial growth of superconducting Sr_1−*x*_La_*x*_CuO_2_ thin films. J. Appl. Phys..

[43] van der Pauw L.J. (1958). A Method of Measuring Specific Resistivity and Hall Effect of Discs of Arbitrary Shape. Philips Res. Rep..

[44] Taylor J.R. (1997). An Introduction to Error Analysis.

[45] Mercone S., Perroni C.A., Cataudella V., Adamo C., Angeloni M., Aruta C., De Filippis G., Miletto F., Oropallo A., Perna P. (2005). Transport properties in manganite thin films. Phys. Rev. B.

[46] Orgiani P., Adamo C., Barone C., Galdi A., Petrov A.Y., Schlom D.G., Maritato L. (2007). Influence of a single disorder parameter on the conduction mechanisms in manganite thin films. Phys. Rev. B.

[47] Mott N.F. (1968). Metal-Insulator Transition. Rev. Mod. Phys..

[48] Yu W., Higgins J.S., Bach P., Greene R.L. (2007). Transport evidence of a magnetic quantum phase transition in electron-doped high-temperature superconductors. Phys. Rev. B.

[49] Fujita M., Matsuda M., Fåk B., Frost C.D., Yamada K. (2006). Novel spin excitations in optimally electron-doped Pr_0.89_LaCe_0.11_CuO_4_. J. Phys. Soc. Jpn..

[50] Ishii K., Fujita M., Sasaki T., Minola M., Dellea G., Mazzoli C., Kummer K., Ghiringhelli G., Braicovich L., Tohyama T. (2014). High-energy spin and charge excitations in electron-doped copper oxide superconductors. Nat. Commun..

[51] Winarsih S., Budiman F., Tanaka H., Adachi T., Koda A., Horibe Y., Kurniawan B., Watanabe I., Risdiana R. (2021). Observation of Cu spin fluctuations in high-T_*c*_ cuprate superconductor nanoparticles investigated by muon spin relaxation. Nanomaterials.

[52] Rivier N., Zlatic V. (1972). Temperature dependence of the resistivity due to localized spin fluctuations. II. Coles alloys. J. Phys. F Met. Phys..

[53] Tallon J.L., Bernhard C., Shaked H., Hitterman R.L., Jorgensen J.D. (1995). Generic superconducting phase behavior in high-T_*c*_ cuprates: T_*c*_ variation with hole concentration in YBa_2_Cu_3_O_7−*δ*_. Phys. Rev. B.

[54] Boato G., Gallinaro G., Rizzuto C. (1966). Effect of Transition-Metal Impurities on the Critical Temperature of Superconducting Al, Zn, In, and Sn. Phys. Rev..

[55] Sipos B., Barisic N., Gaal R., Forró L., Karpinski J., Rullier-Albenque F. (2007). Matthiessen’s rule in MgB_2_: Resistivity and T_*c*_ as a function of point defect concentration. Phys. Rev. B.

